# Early path nursing improves neurological function recovery in patients with intracerebral hemorrhage: Protocol for a randomized controlled trial

**DOI:** 10.1097/MD.0000000000024020

**Published:** 2021-01-08

**Authors:** Yan Yang, Aiping Mu, Yuwen Wang

**Affiliations:** Department of Neurosurgery, The First Affiliated Hospital of Soochow University, Jiangsu, China.

**Keywords:** early path nursing, intracerebral hemorrhage, protocol, quality of life

## Abstract

**Background:**

To explore the influence of the early path nursing on life quality and the neurological function recovery in the intracerebral hemorrhage (ICH) patients.

**Methods:**

The experiment was implemented from January 2018 to October 2020 at the First Affiliated Hospital of Soochow University. The experiment was granted through the Research Ethics Committee of the First Affiliated Hospital of Soochow University (2017033). In this experiment, the criteria for inclusion includes: hemorrhagic stroke diagnosed via the MRI or head CT; over 18 years of age; patients with motor dysfunction; The Glasgow Coma Scale > 12. The patients with these symptoms will be excluded: severe cognitive impairment; ischemic stroke; onset time > 3 days; and severe complications. The scale used for the evaluation the neurological function is the American Stroke Scale. This scale contains a total of eleven items, that is, the movements of upper and lower limb, the consciousness level, gaze, visual field, etc. Other outcomes include patient satisfaction and complications.

**Results:**

Evaluation the neurological function and quality of life will be shown in Table 1.

**Conclusion:**

The early path nursing can promote the neurological function recovery in the ICH patients.

**Trial registration number:**

researchregistry6327

## 1 Introduction

Stroke is the second major cause of death and one of the main causes of disability in the world.^[1–3]^ As life expectancy increases, the stroke burden worldwide is likely to increase, especially in the low-income and middle-income countries.^[4,5]^ Stroke seriously endangers the health and lives of people, and imposes a heavy and huge mental, economic and material burdens on the patients, their families and society.^[6,7]^ Intracerebral hemorrhage (ICH) is the second most prevalent stroke subtype after the ischemic stroke, accounting for about 10% to 20% of all the strokes.^[8,9]^ ICH will occur when the blood vessels in brain parenchyma ruptures.^[10,11]^ ICH can occur in a pre-existing disease complication, for instance, tumor and vascular malformation, and then is called secondary ICH. In the past several years, with the continuous progress and development of medical science, the pathology and etiology of the ICH have been deeply explored. Nevertheless, the nursing care quality remains the key factor affecting the outcomes of ICH.^[12,13]^ Clinical pathway is a concrete process and a kind of standardized work.^[14,15]^ In the nursing process, our department staff develops, adjusts and implements the nursing plans for ICH patients through the rehabilitation teachers and responsible nurses. The personalized nursing plans can improve the rehabilitation outcome and the compliance of patient, as well as make the nurses more reliable in work process. Currently, the early path nursing has seldom been used in the care of patients with ICH. Therefore, we conduct this randomized controlled study protocol to explore the influence of the early path nursing on life quality and the neurological function recovery in ICH patients.

## 2 Methods

The experiment was implemented from January 2018 to October 2020 at the First Affiliated Hospital of Soochow University. The experiment was granted through the Research Ethics Committee of the First Affiliated Hospital of Soochow University (2017033) and recorded in research registry (researchregistry6327). Before the registration, the patients who are recruited receive written informed consent. Sequence of random numbers is generated by a computer. Sequentially numbered sealed opaque envelopes are used for the concealment of random numbers. All the patients participating in this study are randomly divided into control group and nursing group, with 30 patients in each group.

### 2.1 Inclusion and exclusion criteria

In this experiment, the criteria for inclusion includes: hemorrhagic stroke diagnosed via the MRI or head CT; over 18 years of age; patients with motor dysfunction; The Glasgow Coma Scale > 12. The patients with these symptoms will be excluded: severe cognitive impairment; ischemic stroke; onset time > 3 days; and severe complications.

### 2.2 Early path nursing

The patients in both groups are given the routine care. In nursing group, the patients also receive path-based intervention of early rehabilitation nursing. The specific intervention method includes: Intervention time: The patient will be assessed within twenty-four hours after the admission by the medical personnel and nursing staff in charge of the ward. It primarily contains the neurological and physical situations, and the mobility. Meanwhile, medical personnel have set the treatment target and rehabilitation target. Rehabilitation exercise: The rehabilitation training is conducted on the basis of the path chart. On the first day, a detailed assessment of physical function is performed and the patient is assisted in joint activity and physical movement in bed. On the second day, the major task is to teach patient how to turn over. During the process of training, therapists tell the patient to focus on the physical experience and focus on their learning. On the third day, therapists conduct the training of sitting balance and emphasize the cultivation of the self-care ability of patients. On the fourth day, the condition of patient requires to be assessed. It primarily involves the limb flexibility, and the muscle tone and strength of patients. In this exercise, when you are sitting, you need to lean your torso forward, move your center of gravity to the front foot, and the weight is done through the patient's legs. The 5 to 7 days are primarily for the training to stand. Let the patient's feet apart, as wide as the shoulders, and then swing the body to the left and right, mainly to move the center of gravity to the affected side. This process requires attention to protect the patient's knee joint. On the eighth day, muscle tone and strength, and the physical activity of patients are observed. Adjust the treatment approaches appropriately, and then carry on the resistance training of lower limb. From day nine until discharge, focus on the assessment of the motor function of patients and offer some necessary guidance. The responsible nurses should conduct the assessment of daily of patients and take the individualized rehabilitation measures. The rehabilitation therapist implements the weekly consultations on the condition of patients and gives guidance.

### 2.3 Outcomes

The scale used for the evaluation the neurological function is the American Stroke Scale. The scale includes eleven items, namely the level of consciousness, upper and lower limb movements, visual field, gaze, and so on. Other outcomes include patient satisfaction and complications.

### 2.4 Statistical analysis

The analysis of all the data are conducted with the software of IBM SPSS Statistics for Windows, version 20 (IBM Corp., Armonk, NY). Data acquired are represented through the proper features, e.g. standard deviation, and mean, median as well as percentage. And independent t-tests and χ^2^-tests are respectively utilized to analyze the categorical variable and continuous variable. When *P* < .05, the efficacy is viewed to be statistically significant.

## 3 Result

Evaluation of the neurological function and quality of life will be shown in Table 1.

**Table 1:** Evaluation the neurological function and quality of life between two groups.

	Nursing group (n = 30)	Control group (n = 30)	*P* value
American Stroke Scale			
Length of hospital stay			
Total cost			
Complications			
Quality of life			

## 4 Discussion

To our knowledge, this is the first randomized controlled research designed to explore the influence of the early path care on life quality and the neurological function recovery in the ICH patients. Pathway-based early nursing of rehabilitation possesses a positive effect on the recovery of ICH patients’ neurological function. The clinical pathway is a kind of specific process and standardized work whose major activity is to measure the prevalent and multiple diseases.^[16,17]^ The personalized and planned nursing plan can enhance the rehabilitation and compliance of the patient, which can also enable the nursing staff to obtain more information in the process of work, thereby avoiding the blind nursing of the nursing staff. In summary, early path nursing of rehabilitation for ICH patients can enable the nursing staff to provide high-quality nursing services and help the patients recover daily activities and neurological functions as soon as possible.

## 5 Conclusion

The early path nursing can promote the neurological function recovery in the ICH patients.

## Author contributions

**Conceptualization:** Aiping Mu.

**Data curation:** Aiping Mu.

**Funding acquisition:** Yuwen Wang.

**Writing – original draft:** Yan Yang.

## References

[1] Prabhakaran S Ruff I Bernstein RA Acute stroke intervention: a systematic review. JAMA 2015;313:1451–62. 25871671 10.1001/jama.2015.3058

[2] Tchero H Tabue TM Lannuzel A Telerehabilitation for stroke survivors: systematic review and meta-analysis. J Med Internet Res 2018;20:e10867. 30368437 10.2196/10867 PMC6250558

[3] Kurtzke JF Epidemiology of stroke: methods and trends. Health Rep 1994;6:13–21. 7919070

[4] Katan M Luft A Global burden of stroke. Semin Neurol 2018;38:208–11. 29791947 10.1055/s-0038-1649503

[5] Rajsic S Gothe H Borba HH Economic burden of stroke: a systematic review on post-stroke care. Eur J Health Econ 2019;20:107–34. 29909569 10.1007/s10198-018-0984-0

[6] Ramos-Lima M Brasileiro IC Lima TL Quality of life after stroke: impact of clinical and sociodemographic factors. Clinics (Sao Paulo) 2018;73:e418. 30304300 10.6061/clinics/2017/e418 PMC6152181

[7] Mackenzie A Perry L Lockhart E Family carers of stroke survivors: needs, knowledge, satisfaction and competence in caring. Disabil Rehabil 2007;29:111–21. 17364762 10.1080/09638280600731599

[8] Thabet AM Kottapally M Hemphill JR Management of intracerebral hemorrhage. Handb Clin Neurol 2017;140:177–94. 28187799 10.1016/B978-0-444-63600-3.00011-8

[9] An SJ Kim TJ Yoon BW Epidemiology, risk factors, and clinical features of intracerebral hemorrhage: an update. J Stroke 2017;19:3–10. 28178408 10.5853/jos.2016.00864 PMC5307940

[10] Guzik A Bushnell C Stroke epidemiology and risk factor management. Continuum (Minneap Minn) 2017;23:15–39. 28157742 10.1212/CON.0000000000000416

[11] Belanger HG Recovery from stroke: factors affecting prognosis. Clin Neuropsychol 2019;33:813–6. 30882270 10.1080/13854046.2019.1578899

[12] Theofanidis D Gibbon B Nursing interventions in stroke care delivery: an evidence-based clinical review. J Vasc Nurs 2016;34:144–51. 27863592 10.1016/j.jvn.2016.07.001

[13] Clarke DJ Nursing practice in stroke rehabilitation: systematic review and meta-ethnography. J Clin Nurs 2014;23:1201–26. 24102924 10.1111/jocn.12334

[14] Lawal AK Rotter T Kinsman L What is a clinical pathway? Refinement of an operational definition to identify clinical pathway studies for a Cochrane systematic review. BMC Med 2016;14:35. 26904977 10.1186/s12916-016-0580-z PMC4765053

[15] Kinsman L Clinical pathway compliance and quality improvement. Nurs Stand 2004;18:33–5. 14768230

[16] Vanhaecht K De Witte K Depreitere R Clinical pathway audit tools: a systematic review. J Nurs Manag 2006;14:529–37. 17004963 10.1111/j.1365-2934.2006.00705.x

[17] Wang ZE Shangguan QM Wu P Review of the initiation and development of the conception of clinical pathway. Zhonghua Yi Shi Za Zhi 2010;40:341–5. 21223703

